# Hiccups and Acute Symptomatic Hyponatremia: A Rare Manifestation of COVID-19

**DOI:** 10.7759/cureus.24090

**Published:** 2022-04-12

**Authors:** Praveen K Pandey, Divya Pandey, Rebecca Andrews

**Affiliations:** 1 Department of Molecular Medicine, University of Pavia, Pavia, ITA; 2 Internal Medicine Residency Program, University of Connecticut, Farmington, USA

**Keywords:** covid-19, acute symptomatic hyponatremia, persistent hiccups, hyponatremia, hiccups

## Abstract

We report a rare case of a patient presenting with a very unusual manifestation of coronavirus disease 2019 (COVID-19).

A 62-year-old male presented to the emergency department (ED) with complaints of headache, nausea, vomiting, and intractable hiccups for two days. Laboratory results showed that he had profound hyponatremia (Na+: 103 mEq/L) and hypokalemia (K+: 2.3 mEq/L) with a positive RT-PCR for COVID-19. He was treated with an intravenous (IV) bolus of 3% saline solution followed by continuous infusion.

The patient was discharged after eight days when his electrolytes returned to normal, and a significant symptomatic relief was achieved.

## Introduction

Coronavirus disease 2019 (COVID-19) is an infectious disease caused by severe acute respiratory syndrome coronavirus 2 (SARS-CoV-2). The first known case was identified in Wuhan, China, in December 2019. The disease has since spread worldwide, leading to an ongoing pandemic. As of February 13, 2022, over 408 million cases and 5.8 million deaths have been reported globally [1].

Among patients with symptomatic COVID-19, fever, cough, myalgias, and headache are the most commonly reported symptoms. Other features, including diarrhea, sore throat, smell or taste abnormalities, congestion or runny nose, and nausea or vomiting, are also well described [2].

Some patients present with atypical signs and symptoms that are important for an emergency department (ED) or primary care physician to recognize for prompt diagnosis. We present this COVID-19 case report of a patient with a very rare manifestation (hiccups and acute symptomatic hyponatremia).

A preprint of this manuscript was submitted to Research Square (Pandey PK, Pandey D. Hiccups and Acute Symptomatic Hyponatremia: A Rare Manifestation of COVID-19. Research Square. January 31, 2022. doi: 10.21203/rs.3.rs-1252678/v1).

## Case presentation

A 62-year-old male presented to the ED with complaints of headache, nausea, vomiting, and a history of persistent hiccups for the last two days. He reported that the hiccups started suddenly and increased in intensity over time, leading to mild dyspnea and difficulty eating. Vital signs revealed a SpO2 of 98% on room air and a blood pressure reading of 149/89 mm Hg. The patient reported no history of fever, coughs, or any other chronic health conditions. Volume status was accessed, and the patient was found to be clinically euvolemic, with no signs of either volume depletion (tachycardia, dry mucous membranes, decreased skin turgor, or orthostasis) or excessive volume of extracellular fluid (edema or ascites).

Laboratory tests for liver function, renal function, complete blood count, and serum electrolytes were performed in conjunction with an RT-PCR for COVID-19 (as per local guidelines).

The patient had profound hyponatremia (Na+: 103 mEq/L) and hypokalemia (K+: 2.3 mEq/L).

RT-PCR was positive for COVID-19. The patient was treated with a 100 mL intravenous (IV) bolus of 3% NaCl over 10 minutes and admitted to the COVID ICU where he was treated with an intravenous infusion of 3% NaCl (10 mL/kg/hour) with frequent monitoring of serum sodium along with charting of input (restricted to 1200 mL/day) and output.

The patient had occasional relief from hiccups as the serum Na+ levels improved but displayed marked forgetfulness (unable to remember if he had eaten a meal or not).

Additional tests for C-reactive protein (34 mg/L), D-dimer (903 ng/mL), lactate dehydrogenase (LDH) (174 IU/L), N-terminal pro-B-type natriuretic peptide (NT-proBNP) (44 pg/mL), procalcitonin (0.089 ng/mL), and abdominal ultrasound (no significant abnormality) to rule out other causes of SIADH were ordered.

A summary of all laboratory investigations of the patient is presented in Table 1.

**Table 1:** Summary of all laboratory investigations of the patient. TLC: total leukocyte count, AST: aspartate transaminase, ALT: alanine transaminase, LDH: lactate dehydrogenase, NT-proBNP: N-terminal pro-B-type natriuretic peptide

Laboratory tests	Results	Units	Biological reference ranges
Hemoglobin	14.4	g/dL	12-15
Platelet count	165	thousand/mm^3^	150-410
RBC count	4.66	million/mm^3^	3.8-8.8
TLC	38	thousand/mm^3^	4-10
Urea	17	mg/dL	13-43
Creatinine	1.2	mg/dL	0.7-1.3
Total bilirubin	0.7	mg/dL	0.3-1.2
Conjugated bilirubin	0.2	mg/dL	<0.3
Unconjugated bilirubin	0.5	mg/dL	<1.1
AST (SGOT)	40	IU/L	15-40
ALT (SGPT)	33	IU/L	10-49
Albumin	3.3	mg/dL	3.2-4.8
Calcium	0.56	mmol/L	2-2.6
Sodium	103	mEq/L	136-145
Potassium	2.3	mEq/L	3.5-5.1
C-reactive protein	34	mg/L	<5
D-dimer	903.5	ng/mL	<500
LDH	174	IU/L	<248
Procalcitonin	0.089	ng/mL	<0.5
NT-proBNP	44	pg/mL	<115

After eight days, as serum electrolyte values moved gradually but persistently toward normalcy (Na+: 131 mEq/L and K+: 3.6 mEq/L; after 12 hours, Na+: 134 mEq/L and K+: 3.5 mEq/L), the patient had significant symptomatic relief from hiccups and was able to ingest both solid and liquid foods without any discomfort.

A repeat RT-PCR for COVID-19 also came back negative. The patient was discharged to follow-up with his outpatient physician in one week.

## Discussion

A hiccup, or singultus, is an involuntary spastic contraction of the inspiratory muscles. Although usually a benign and self-limited annoyance, persistent hiccups may be a sign of serious underlying illness.

The exact pathogenesis of hiccups remains unknown. In most cases in which a specific cause can be assigned, hiccups appear to result from stimulation, inflammation, or injury to one of the nerves of the hiccup reflex arc.

SARS-CoV-2 has been known to show selective neurotropism to areas of the brain controlling respiration [3], which also control the hiccup reflex arc. Irritation or stimulation of this neural area can be a plausible explanation for hiccups in COVID-19. Several case reports have been published [4-10] that report hiccups as a symptom of COVID-19, and one study proposes hiccups as a specific neurological symptom in males [11].

Hyponatremia is defined as a serum sodium concentration of less than 135 mEq/L and is the most common electrolyte abnormality encountered in clinical practice. The Joint European guidelines classify hyponatremia in adults according to serum sodium concentration as follows: mild, 130-134 mEq/L; moderate, 125-129 mEq/L; and profound, <125 mEq/L. Acute symptomatic hyponatremia is a medical emergency. A sudden drop in serum Na+ can overwhelm the capacity of the brain to regulate cell volume, leading to cerebral edema, seizures, and death. Hyponatremia, both asymptomatic and acute symptomatic, has been reported to be associated with COVID-19 in many case reports [12-14].

Hyponatremia in COVID-19 can be caused by either SIADH or inflammatory cytokines, IL-6, or a combination [15].

To the best of our knowledge, this is the first case that reports both acute symptomatic hyponatremia and hiccups present simultaneously in a COVID-19 patient. Hiccups and hyponatremia can develop in a COVID-19 patient independently; however, hiccups can be triggered by many metabolic disorders, hyponatremia being one [16]. It is difficult to say whether the hyponatremia developed first, which precipitated hiccups, or both hiccups and hyponatremia developed independently. Resolution of hiccups with improvement in serum sodium levels points more toward metabolic etiology as the cause.

## Conclusions

It’s been more than two years since the emergence of COVID-19, and physicians are discovering new symptoms and correlations with possible causations. This case report attempts to document a combination of such rare signs and symptoms in COVID-19, thereby raising the index of suspicion at the point of the ED triage or primary physician for COVID-19 in patients presenting with these manifestations.
